# Spatiotemporal prevalence and characterization of the lineage I insect-specific flavivirus, Quang Binh virus, isolated from Culex gelidus mosquitoes in Singapore

**DOI:** 10.1099/jgv.0.002105

**Published:** 2025-06-02

**Authors:** Christopher Chong Wei Ang, Angelica Ting Yi Ang, Jerald Yam, Rou Xuan Lee, Ming Jie Lim, Zhi Yang Loh, Majhalia Torno, Luqman Hakim, Judith Chui Ching Wong, Jessica J. Harrison, Jody Hobson-Peters, Cheong Huat Tan, Yin Xiang Setoh

**Affiliations:** 1 School of Chemistry and Molecular Biosciences, The University of Queensland, St. Lucia, Queensland, Australia; 2 Environmental Health Institute, National Environment Agency, 11 Biopolis Way, Helios Block, #06-05/08, Singapore 138667, Singapore; 3 Infectious Diseases Translational Research Programme (ID TRP), Yong Loo Lin School of Medicine, National University of Singapore, Singapore, Singapore

**Keywords:** insect-specific flavivirus, Quang Binh virus, spatiotemporal prevalence, superinfection exclusion

## Abstract

Insect-specific flaviviruses (ISFs) are a group of orthoflaviviruses that can replicate efficiently in arthropods but are unable to replicate in vertebrate hosts. This contrasts with medically important orthoflaviviruses, such as dengue virus (DENV) and West Nile virus (WNV). Using the established monoclonal antibody against viral RNA intermediates in cell assay, we report the first isolation of an ISF, Quang Binh virus (QBV), in Singapore from three pools of *Culex gelidus* mosquitoes. To determine the spatiotemporal prevalence of QBV, a total of 17,070 mosquitoes, represented as 721 pools, were screened using a QBV-specific reverse transcriptase quantitative PCR assay, revealing 36 QBV-positive pools of mosquitoes detected mainly along the northern coastal regions of Singapore. Repeated detections over 12 months in a north-western nature reserve suggest local stable establishment of the virus. Phylogenetic and molecular analyses show that QBV sequences from the Singapore group together with other Southeast Asian sequences and that *Cx. gelidus*-derived sequences are phylogenetically distinct from those derived from *Culex tritaeniorhynchus*. We also present *in vitro* evidence that QBV is able to suppress DENV2 and WNV_KUN_ in C6/36 cells by 2.9 logs and 1.8 logs, respectively. This report represents the first known spatiotemporal study of an ISF and highlights QBV’s potential as a biological control against medically important orthoflaviviruses.

## Data Availability

Quang Binh virus full genome sequences have been deposited in GenBank under accession numbers PV010628–PV010630. Quang Binh virus partial NS5 sequences have been deposited in GenBank under accession numbers PV010631–PV010635.

## Introduction

Arthropod-borne viruses (arboviruses) are transmitted through the bite of an infected haematophagous arthropod to a vertebrate host. Notably, members of the genus *Orthoflavivirus* such as dengue virus (DENV), Japanese encephalitis virus (JEV), West Nile virus (WNV), yellow fever virus and Zika virus, as well as the *Alphavirus*, chikungunya virus, have been associated with arboviral disease epidemics in the past five decades. These arboviruses are transmitted by either *Aedes* spp. or *Culex* spp. mosquitoes as their arthropod vectors [1,2].

Orthoflaviviruses are enveloped viruses containing a non-segmented, positive-strand ssRNA (+ssRNA) genome of ~11 kb. The genome consists of an ORF encoding a single polyprotein that is co- and post-translationally cleaved into three structural proteins (C, prM and E) and seven non-structural proteins (NS1, NS2A, NS2B, NS3, NS4A, NS4B and NS5). The ORF is flanked by 5′ and 3′ UTRs [3]. Orthoflaviviruses can be grouped according to their host range: arthropod-borne, also known as dual-host orthoflaviviruses, vertebrate-specific (no known vector) and insect-specific flaviviruses (ISFs) [4,5].

As their name suggests, ISFs can replicate efficiently in arthropods but are unable to replicate in vertebrate hosts [4]. ISFs can be further divided into two paraphyletic groups. Classical ISFs, also called lineage I ISFs, are phylogenetically distinct from other orthoflaviviruses, while dual-host affiliated ISFs, also called lineage II ISFs, are more closely related to vertebrate-infecting flaviviruses (VIFs) [4]. Phylogenetic analysis suggests that lineage I ISFs are ancestral to VIFs and can thus provide insights into the expanded host range and transmission mechanisms of arthropod-borne orthoflaviviruses [6]. Cell-fusing agent virus (CFAV), a lineage I ISF, was the first ISF to be discovered in *Aedes aegypti* cell cultures back in 1975 [7]. A gap of almost 30 years followed before the discovery of Kamiti River virus, another lineage I ISF [8,9]. With the advancement of molecular-based detection assays and an increasing interest in insect-specific viruses (ISVs), there has been an exponential increase in ISF discovery over the past 15 years [10,37].

Due to their unique host range-restricted phenotype, various studies have been done to investigate the potential biotechnological applications of ISFs. Superinfection exclusion, a phenomenon where the secondary infection of a virus is inhibited by the primary infection of another virus, has been observed for a handful of ISFs *in vitro* and *in vivo* [38,44]. This underscores their potential as biological controls against arboviruses, though results across different ISFs have been varied. More recently, chimaeric lineage II ISFs have been generated as potential vaccine candidates and diagnostic antigens [45,51].

In Asia, there have been multiple detections of a lineage I ISF, Quang Binh virus (QBV), which was first discovered in Vietnam in 2009 [12]. Originally isolated from a pool of *Culex tritaeniorhynchus* mosquitos, QBV was shown to replicate in *Aedes* spp. cells but not in a range of mammalian cell lines [12]. Recurrent QBV detections have been reported throughout Asia in *Cx. tritaeniorhynchus*, *Cx. pipiens*, *Cx. gelidus* and *Anopheles sinensis* mosquitos [52,57]. Although ISFs have been thought to adapt with their mosquito host, two separate detections in *An. sinensis* suggest QBV might not be limited to *Culex* spp. mosquitos [52,54]. Of note, Yunnan *Culex* flavivirus (YNCxFV) has an 83% nucleotide sequence identity with QBV, just under the threshold criterion of 84% for a species [52,58]. As such, the classification of YNCxFV as a novel ISF species remains unresolved.

Previously, an orthoflavivirus with 80% nucleotide sequence identity to QBV was detected, but not isolated, in Singapore, prompting us to investigate the prevalence of ISFs in the country [59]. In this study, we report the first isolation of an ISF, QBV, from three pools of *Cx. gelidus* mosquitoes in Singapore using the monoclonal antibodies against viral RNA *i*ntermediates in cells (MAVRIC) assay [60,61]. The goal of this study was thus to investigate the spatiotemporal prevalence of the ISF locally and assess its potential as a biological control tool against other medically important orthoflaviviruses.

## Methods

### Mosquito collection, identification and processing

**Fig. 1.**
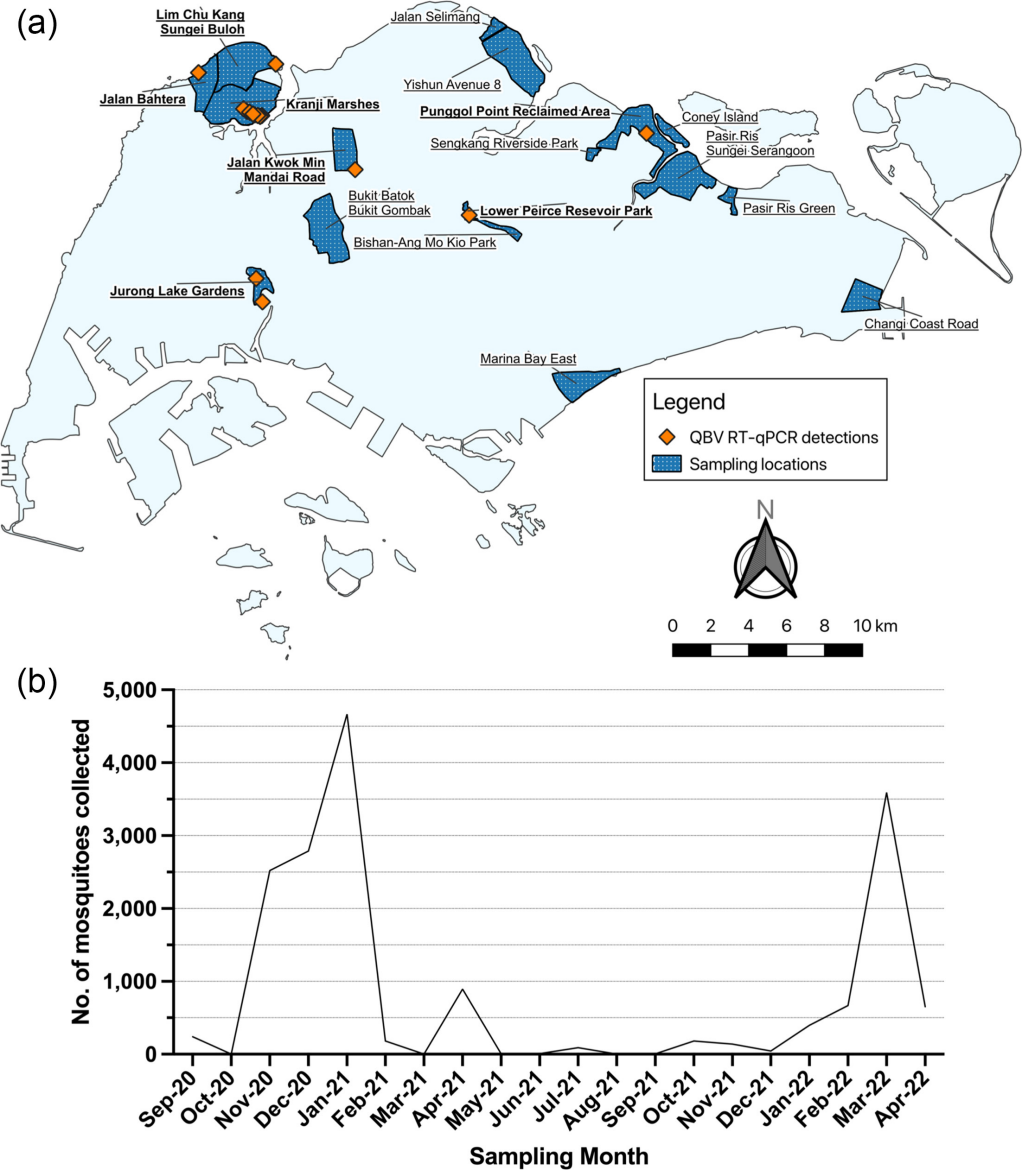
Mosquitoes collected from September 2020 to April 2022. (**a**) Map of mosquito sampling locations. Each sampling location, shown as dark blue polygons, contains one to nine trapping sites. Trapping sites with QBV detected are shown as orange diamonds, while sampling locations with QBV detections are in bold. (**b**) Temporal distribution of mosquitoes collected.

Mosquitoes were obtained from two National Environment Agency (NEA) surveillance networks across 17 locations (Fig. 1a and Table S1, available in the online Supplementary Material). From the malaria surveillance network, 11,273 mosquitoes were trapped in 12 locations in Singapore from September 2020 to February 2021. Using NEA Night Catcher traps, mosquitoes were trapped hourly between 7 pm and 7 am and transported to the Environmental Health Institute (EHI). Mosquitoes were identified using relevant mosquito identification keys and stored at −20 °C [62,67]. Mosquitoes were pooled by species into tubes of 25–30 specimens on dry ice, followed by the addition of a 4 mm stainless steel bead and 1.5 ml of 2% FBS L-15 Leibovitz or M199 media (Cytiva). Mosquito pools were homogenized at 1,500 r.p.m. for 4 min using the 1600 MiniG Tissue Homogenizer (SPEX SamplePrep), centrifuged at 14,680 r.p.m. for 3 min and filtered through a 0.8/0.22 µm double filter (Pall Corporation). Homogenates were stored at −80 °C until use. RNA was extracted with MagMAX Viral/Pathogen kit (Thermo Scientific) according to the manufacturer’s protocol with 200 µl sample input and three wash steps.

From the arbovirus surveillance network, 5,797 mosquitoes were trapped from 5 different locations in Singapore from April 2021 to April 2022. Trapping, identification and processing followed the previously described methods [68]. Briefly, mosquitoes were trapped over 16–20 h using Centers for Disease Control (CDC) light traps and transported to EHI on dry ice. Mosquitoes were identified using relevant mosquito identification keys and pooled by species into tubes containing up to 100 specimens before storing at −80 °C [62,67]. Mosquito pools were homogenized in 500 µl of universal transport media (Copan Diagnostics) at 5,000 ***g*** for 5 min using the 1600 MiniG Tissue Homogenizer (SPEX SamplePrep) and stored at −80 °C until use. Viral RNA was extracted using the IndiMag Pathogen Kit (Indical Bioscience) following the manufacturer’s protocol. *Culex* spp. pools were screened for JEV using a previously established SYBR Green-based duplex reverse transcriptase quantitative PCR (RT-qPCR) method [59]. JEV-positive samples were excluded from further processing.

### Cell and virus culture

C6/36 cells (ATCC CRL-1660) were grown in L-15 Leibovitz media containing l-glutamine (Cytiva) supplemented with 10% FBS, 2 mM l-glutamine, 1 mM sodium pyruvate, 100 U ml^−1^ penicillin and 100 μg ml^−1^ streptomycin. African Green Monkey Kidney cells (Vero, ATCC CCL-81) were grown in Medium 199 containing Earle’s balanced salt solution and l-glutamine (Cytiva) supplemented with 10% FBS, 2 mM l-glutamine, 1 mM sodium pyruvate, 10 mM HEPES buffer, 100 U ml^−1^ penicillin and 100 μg ml^−1^ streptomycin. Baby hamster kidney cells (BHK-21, ATCC CCL-10) were grown in RPMI 1640 media containing l-glutamine (Cytiva) supplemented with 5% FBS, 2 mM l-glutamine, 1 mM sodium pyruvate, 10 mM HEPES buffer, 100 U ml^−1^ penicillin and 100 μg ml^−1^ streptomycin. C6/36 cells were cultured at 28 °C with 0% CO_2_, while mammalian cells were cultured at 37 °C with 5% CO_2_. ISV stocks were propagated by inoculating onto C6/36 monolayers with 2% FBS L-15 Leibovitz media and incubated at 28 °C with 0% CO_2_. Supernatants were harvested after 5–7 days and stored at −80 °C. Stocks were titrated via serial 10-fold dilutions in 2% FBS L-15 Leibovitz media and inoculated onto C6/36 monolayers in 96-well plates. After 4–5 days, culture supernatants were removed, and cells were fixed with 4% formaldehyde containing 0.5 % v/v Triton X-100 for 10 min at 4 °C. Plates were air dried for 4–7 days before MAVRIC-ELISA was carried out using a 1:1 3G1 and 2G4 cocktail as previously described [61]. MAVRIC-ELISA uses anti-dsRNA 3G1 and 2G4 antibodies and has been shown to detect a range of +ssRNA and dsRNA viruses [60,61]. Viral titres were determined as TCID_50_ using the method described by Reed and Muench [69].

### Virus isolation

C6/36 cells were seeded at 5×10^4^ cells/well in 96-well plates 2 days before infection. For each sample, 25 µl of filtered mosquito homogenates from the malaria surveillance network was inoculated onto cell monolayers in quadruplicate and incubated at 28 °C with 0% CO_2_ for 2 h. The inoculum was replaced with 100 µl/well of 2 % FBS L-15 Leibovitz media, and plates were incubated for 4–7 days at 28 °C with 0% CO_2_. Culture supernatants were harvested and blind passaged onto new 96-well plates seeded with C6/36 cells. MAVRIC-ELISA was performed on infected cell monolayers after each blind passage as previously described [60,61]. Briefly, cells were fixed with 4% formaldehyde containing 0.5% v/v Triton X-100 for 10 min at 4 °C and then air dried for 4–7 days. Plates were blocked with 150 µl/well of Pierce Protein-Free Blocking Buffer (Thermo Scientific) containing 0.05 % v/v Tween 20 for 1 h at 37 °C. Wells were then probed with 50 µl/well 3G1 and 2G4 (1:1) antibodies (Mozzy Mabs), which are monoclonal antibodies against dsRNA, diluted 1:64 with blocking buffer for 1 h at 37 °C, followed by four washes with phosphate-buffered saline containing 0.05 % v/v Tween 20 (PBST). Next, 50 µl/well of goat anti-mouse Ig/HRP antibody (DAKO) diluted 1:800 in blocking buffer was added and incubated for 1 h at 37 °C before six washes with PBST. Next, 150 µl/well of one-step 2,2′-azinobis [3-ethylbenzothiazoline-6-sulfonic acid]-diammonium salt substrate solution (Thermo Scientific) was added and incubated in the dark at room temperature for 2 h. Absorbance was measured at 405 nm, with wells considered positive if absorbance values exceeded twice the negative control wells.

### Reverse transcriptase PCR and Sanger sequencing

RNA was extracted from 140 µl of culture supernatants using the QIAamp Viral RNA Mini Kit (QIAGEN). For MAVRIC-positive cultures, reverse transcriptase PCR (RT-PCR) was performed using the Superscript III One-Step RT-PCR System with Platinum *Taq* DNA Polymerase (Thermo Scientific) using pan-orthoflavivirus (FU2 : 5′-GCTGATGACACCGCCGGCTGGGACAC-3′ and cFD3: 5′-AGCATGTCTTCCGTGGTCATCCA-3′) and generic mesonivirus (ISVs belonging to the order *Nidovirales*) (NidoF1 : 5′-GTTGTATGCTATGCCGYCG-3′ and NidoR1 : 5′-TCCATAGTATCGTAGCAATTCC-3′) primers with the following conditions: reverse transcription (RT), 45 °C/15 min and 50 °C/15 min, and PCR, 1 cycle 94 °C/2 min, 40 cycles 94 °C/30 s, 45 °C/30 s and 68 °C/45 s, followed by a final extension of 68 °C/5 min [58,70]. For QBV cultures, sequencing primers were designed in-house by aligning five QBV whole-genome sequences from the NCBI with the partial NS5 amplicon obtained from QBV isolate 490 Cx. ge 9/2/21 T1 (QBV_seq_1F: 5′-TGGGACACTAAGATCACGGAAGC-3′ and QBV_seq_1R: 5′-ACGATGGCAGCGAATCCTAG-3′) using clustalw [71]. Two-step RT-PCR was performed on RNA from QBV-positive mosquito pool homogenates to generate amplicons for Sanger sequencing. Briefly, cDNA was generated using the Maxima H Minus First Strand cDNA Synthesis kit (Thermo Scientific) with random primers as per the manufacturer’s instructions. PCR was performed with Phusion Flash High-Fidelity PCR Master Mix (Thermo Scientific) using QBV sequencing primers with the following conditions: 1 cycle 98 °C/10 s and 40 cycles 98 °C/5 s, 55 °C/10 s and 72 °C/30 s, followed by a final extension of 72 °C/1 min. PCR amplicons were excised, and DNA was extracted using the Monarch DNA Gel Extraction Kit (New England Biolabs) as per the manufacturer’s instructions before sending it to the first BASE for Sanger sequencing.

### Next-generation sequencing

Next-generation sequencing (NGS) was used to identify unknown viruses (MAVRIC positive for dsRNA but PCR negative for orthoflavivirus and mesonivirus RNA) via *de novo* sequencing and to obtain full genome sequences of QBV via multiplex PCR. For unknown virus identification, 10 ml of culture supernatant was concentrated using Amicon Ultra Centrifugal 30 kDa Filter tubes (Sigma-Aldrich) by centrifugation at 3,000 r.p.m. for 20 min. RNA was extracted from 140 µl of concentrated supernatant using the QIAamp Viral RNA Mini Kit (QIAGEN) without carrier RNA following the manufacturer’s protocol. Sequence-independent, single-primer amplification (SISPA) was performed as previously described [72]. PCR products were purified with AMPure XP beads (Beckman Coulter) at a 1.0x ratio before 2×150 bp sequencing on the NovaSeq 6000 platform. Adapter sequences were trimmed with Trimmomatic v0.39, and SISPA ‘8N’ sequences were removed with cutadapt v1.18 [73,74]. *In silico* host-read removal was performed with bowtie2 v2.3.5.1 and SAMtools v1.16.1 using the *Aedes albopictus* C6/36 genome (NCBI RefSeq assembly: GCF_001876365.2) as the reference [75,76]. *De novo* assembly of the host-filtered reads was performed with SPAdes v3.15.2 [77]. Contigs with >10× coverage were subjected to blast analysis for viral sequence identification. Full genome sequencing of QBV was performed using multiplex PCR with 800 bp amplicons designed via primalscheme (primalscheme.com) as previously described [78]. Primers targeting the 5′ and 3′ UTRs were designed based on complete QBV genomes from the NCBI. Amplicons were generated using two-step RT-PCR, as described above, and purified using AMPure XP beads (Beckman Coulter) at a 1.8x ratio before 2×150 bp sequencing on the NovaSeq 6000 platform. Raw reads were assessed with FastQC v0.12.1 (http://www.bioinformatics.babraham.ac.uk/projects/fastqc/), and Illumina adapter sequences were trimmed with Trimmomatic v0.39 [73]. Trimmed paired-end reads were mapped to the reference genome using bwa-mem2 v2.2.1 and further processed with SAMtools v1.16.1 [76,79]. iVar v1.4 was used to trim primer sequences from the reads using a BED file with the primer positions, followed by further quality trimming and finally consensus sequence generation with a combination of SAMtools and iVar [80]. A complete list of QBV sequencing primers and sequences used in this study is available in Tables S2 and S3, respectively.

### RT-qPCR screening

QBV-specific primers and probes were designed in-house using PrimerQuest (IDT) from the partial NS5 sequence obtained from RT-PCR (QBV_SG_F4 : 5′-TTCTGCTCCCACCATTTCC-3′, QBV_SG_R4 : 5′-CCGAGCTCTCCCAATGATTT-3′ and QBV_SG_Prb4 : 5′-6-FAM-TGCTTAGAGACGGACGAGAGATCA-BHQ1-3′). RT-qPCR was performed using the Luna Universal Probe One-Step RT-qPCR kit (New England Biolabs) as per the manufacturer’s instructions. Briefly, reactions were set up with 0.4 µM primers, 0.2 µM probes and 2.5 µl of RNA template in a final reaction volume of 20 µl. The assay was run on QuantStudio 5 (Thermo Scientific) using the following conditions: RT, 55 °C/10 min; quantitative PCR: 1 cycle 95 °C/1 min; 45 cycles 95 °C/10 s, 60 °C/30 s+plate read. Primer and probe specificity for QBV was confirmed via a blast (NCBI) search and RT-qPCR cross-reactivity testing against DENV and WNV_KUNV_ isolate RNA. Following MIQE guidelines, the assay was determined to have a PCR efficiency of 93.3% [81]. Mosquito pools were considered QBV RT-qPCR positive if *C*_*t*_ ≤39.7.

### Sequence alignments and phylogenetic analysis

Sequences obtained in this study were compared with 14 full and partial QBV genome sequences available from the NCBI. YNCxFV (KC464457.1) and Yamadai flavivirus (AB981186.1) were used as outgroups. Multiple sequence alignments were performed using clustalw and analysed with Molecular Evolutionary Genetics Analysis (mega) [71,82]. Phylogenetic trees were constructed with IQ-TREE using the maximum-likelihood method and 1,000 replicates [83]. The TNe+I model was applied for partial NS5 trees, while the GTR+F+G4 model was used for complete coding sequence trees.

### Viral growth kinetics

All cells were seeded in six-well plates. Vero cells were seeded at 4×10^5^ cells 1 day before infection, while C6/36 cells were seeded at 1×10^6^ cells 2 days before infection. Cell monolayers were either infected with QBV at an m.o.i. of 0.1 or mock infected with 2% FBS media. Cells were washed once with PBS, and 500 µl/well of inoculum was added. Vero cells were incubated at 37 °C with 5% CO_2_, while C6/36 cells were incubated at 28 °C with 0 % CO_2_ for 1 h, rocking every 15 min. Following infection, cell monolayers were washed once with PBS, and 2 ml/well of 2% FBS media was added. Cells were incubated in the same conditions described above. At each time point, 250 µl of culture supernatant was harvested and stored at −80 °C. Viral titres were determined using MAVRIC-ELISA, as described above.

### Superinfection exclusion experiments

C6/36 cells were seeded in 6-well plates at 9×10^5^ cells 2 days before infection. Cells were washed once with sterile PBS and infected with 500 µl/well of QBV at an m.o.i. of 1. For mock-infected wells, this was done with 2% FBS L-15 Leibovitz media instead. Plates were incubated at 28 °C with 0% CO_2_ for 1 h, rocking every 15 min. The inoculum was replaced with 2 ml/well of 2% FBS L-15 Leibovitz media, and cells were allowed to incubate for 1 h at 28 °C with 0% CO_2_. Cells were then inoculated with DENV2 (strain EHIE51858Y19, Cosmopolitan genotype, passage 3) or WNV_KUN_ (strain MRM61C, passage 3) at an m.o.i. of 0.1 as described in the above viral growth kinetics method, and viral titres were determined using immuno-plaque assay instead of MAVRIC-ELISA.

### Immuno-plaque assay

BHK-21 (CCL-10) and Vero (CCL-81) cells were used to titre DENV2 and WNV_KUN_, respectively, using media as described above. 2×10^4^ cells were seeded in 96-well plates 1 day before infection. Culture supernatants were diluted via 10-fold serial dilutions in 2% FBS media, and 25 µl of each dilution was inoculated onto confluent monolayers. Plates were incubated at 37 °C for 1 h. The immuno-plaque assay was conducted as previously described by Goh *et al.* [84]. Briefly, 150 µl overlay media (2x media supplemented with 4% FBS, 7.5 % w/v sodium bicarbonate, 2 mM l-glutamine, 1 mM sodium pyruvate, 100 U ml^−1^ penicillin and 100 μg ml^−1^ streptomycin mixed 1:1 v/v with 2% carboxymethyl cellulose) were added to each well and incubated at 37 °C. Incubation times were virus dependent (DENV2: 40 h post-inoculation, WNV_KUN_: 24 h post-inoculation). After incubation, overlay media were removed, and 200 µl/well of ice-cold 80% acetone was added before fixing at −20 °C for 1 h. Plates were washed six times with PBST and left to dry for 1–3 days. Plates were blocked with 50 µl/well Pierce Protein-Free Blocking Buffer (Thermo Scientific) containing 0.05 % v/v Tween 20 for 1 h at 37 °C. They were then probed with 50 µl/well mAB 4G2 diluted 1:20 with blocking buffer for 1 h at 37 °C, followed by washing four times with PBST. In each well, 50 µl of IRDye 800CW goat anti-mouse antibody (LI-COR) diluted 1:2,000 in blocking buffer was added and incubated for 1 h at 37 °C, followed by washing six times with PBST. Plates were either left to dry overnight in the dark or immediately scanned using the LI-COR Biosciences Odyssey Infrared Imaging System.

## Results

### Detection and isolation of ISVs in mosquitoes

A total of 11,273 mosquitoes, represented as 372 pools, across 12 locations were obtained from EHI’s malaria surveillance network. Mosquitoes from six different genera were screened using the MAVRIC assay to detect dsRNA in cells, including *Aedeomyia* (one species), *Anopheles* (two species), *Coquillettidia* (one species), *Culex* (five species), *Mansonia* (two species) and *Verrallina* (one species) (Table S1). The majority of mosquitoes obtained were *Cx. tritaeniorhynchus* (72.1%), and the highest number of mosquitoes was trapped from Pasir Ris/Sungei Serangoon (*n*=5,280). The NEA Night Catcher, a specialized trap designed to capture nocturnal mosquitoes (i.e. *Culex* spp.), explains the predominance of *Culex* spp. in our sample collection. This trap combines visual stimuli (light-emitting diodes) and chemical attractants (carbon dioxide) to effectively target various mosquito species that are active at night. However, it is less effective at targeting day-biting species, as they typically respond to different environmental cues and attractants.

To minimize false negatives due to the assay’s detection limit, all samples underwent two blind passages. One *Coquillettidia crassipes* and six *Cx. gelidus* mosquito pools were MAVRIC positive at both the first and second passages, indicating the presence of viral dsRNA intermediates. Culture supernatants from these pools were then inoculated onto both C6/36 and Vero cells for a third blind passage to assess the host range of these viruses. All seven pools were MAVRIC positive in C6/36 cells but were MAVRIC negative in Vero cells, indicating that these isolates are ISVs (Table 1). RT-PCR with pan-orthoflavivirus (FU2/cFD3) and generic mesonivirus (NidoF1/NidoR1) primer sets was used in an attempt to identify these virus isolates [58,70]. Only one pool (490 Cx. ge 9/2/21 T1) was RT-PCR positive with pan-orthoflavivirus primers. None were positive for mesonivirus using generic mesonivirus primers. Sequencing of the FU2/cFD3 amplicon revealed >95 % nucleotide sequence identity to QBV isolates from Vietnam and Guangdong. As amplicons were inconsistently produced using FU2/cFD3 primers, a QBV-specific primer set (QBV_seq_1F/QBV_seq_1R) nested within the FU2/cFD3 target was designed. RT-PCR with QBV-specific primers was conducted on all seven MAVRIC-positive pools, revealing two more QBV isolates (490 Cx. ge 9/2/21 T5 and 490 Cx. ge 9/2/21 T6). Sequencing of all three QBV amplicons revealed >96% nucleotide sequence identity to the same Vietnam and Guangdong QBV isolates.

**Table 1.** Summary of MAVRIC-positive mosquito pools

Mosquito species	Tube no.	Pool ID	Mosquito per pool	Date collected	Location	Passage 3 MAVRIC result		Virus identity
						C6/36	Vero	
*Cx. gelidus*	1	490 Cx. ge 9/2/21 T1	30	9 February 2021	Lim Chu Kang/Sungei Buloh	+	−	QBV
*Cx. gelidus*	2	490 Cx. ge 9/2/21 T2	30	9 February 2021	Lim Chu Kang/Sungei Buloh	+	−	Unknown
*Cx. gelidus*	3	490 Cx. ge 9/2/21 T3	30	9 February 2021	Lim Chu Kang/Sungei Buloh	+	−	Unknown
*Cx. gelidus*	4	490 Cx. ge 9/2/21 T4	30	9 February 2021	Lim Chu Kang/Sungei Buloh	+	−	Unknown
*Cx. gelidus*	5	490 Cx. ge 9/2/21 T5	30	9 February 2021	Lim Chu Kang/Sungei Buloh	+	−	QBV
*Cx. gelidus*	6	490 Cx. ge 9/2/21 T6	30	9 February 2021	Lim Chu Kang/Sungei Buloh	+	−	QBV
*Coquillettidia crassipes*	1	377 Cq. cra 8/1/21 T1	30	8 January 2021	Yishun Ave 8	+	−	Sarawak virus

+, A positive sample where one or more infected wells had an optical density (OD) greater than twice the average OD of negative control wells. −, A negative sample where no infected wells had an OD greater than twice the average OD of negative control wells.

To identify the remaining unknown viruses (MAVRIC positive but negative for orthoflavivirus and mesonivirus RNA), NGS and *de novo* assembly were performed. Using this approach, we obtained a near-complete viral genome sequence that has 92% nucleotide identity to Sarawak virus (Family: *Alphatetraviridae*) from the pool of *Coquillettida crassipes* (377 Cq. cra 8/1/21 T1).

### Spatiotemporal prevalence of QBV in Singapore

An additional 5,797 mosquitoes were obtained from EHI’s arbovirus surveillance to determine QBV’s prevalence in Singapore. In total, 17,070 mosquitoes, represented as 721 pools, across 17 locations obtained from September 2020 to April 2022, were screened with a QBV-specific RT-qPCR assay. Samples consisted of seven different genera, including *Aedeomyia* (one species), *Aedes* (one species), *Anopheles* (two species), *Coquillettidia* (one species), *Culex* (one subgenus and eight confirmed species), *Mansonia* (two species) and *Verrallina* (one species) (Table S1). The majority of mosquitoes obtained were *Cx. tritaeniorhynchus* (58.7%), and the highest number of mosquitoes was trapped from Kranji Marshes (*n*=5,323). Throughout the duration of surveillance, mosquito trappings peaked in January 2021 (*n*=4,667) and March 2022 (*n*=3,591) (Fig. 1b). The surge in mosquito collection during these months was mainly contributed by Pasir Ris/Sungei Serangoon (*n*=2,087) and Kranji Marshes (*n*=3,536) sampling locations, respectively (Fig. S1).

**Fig. 2.**
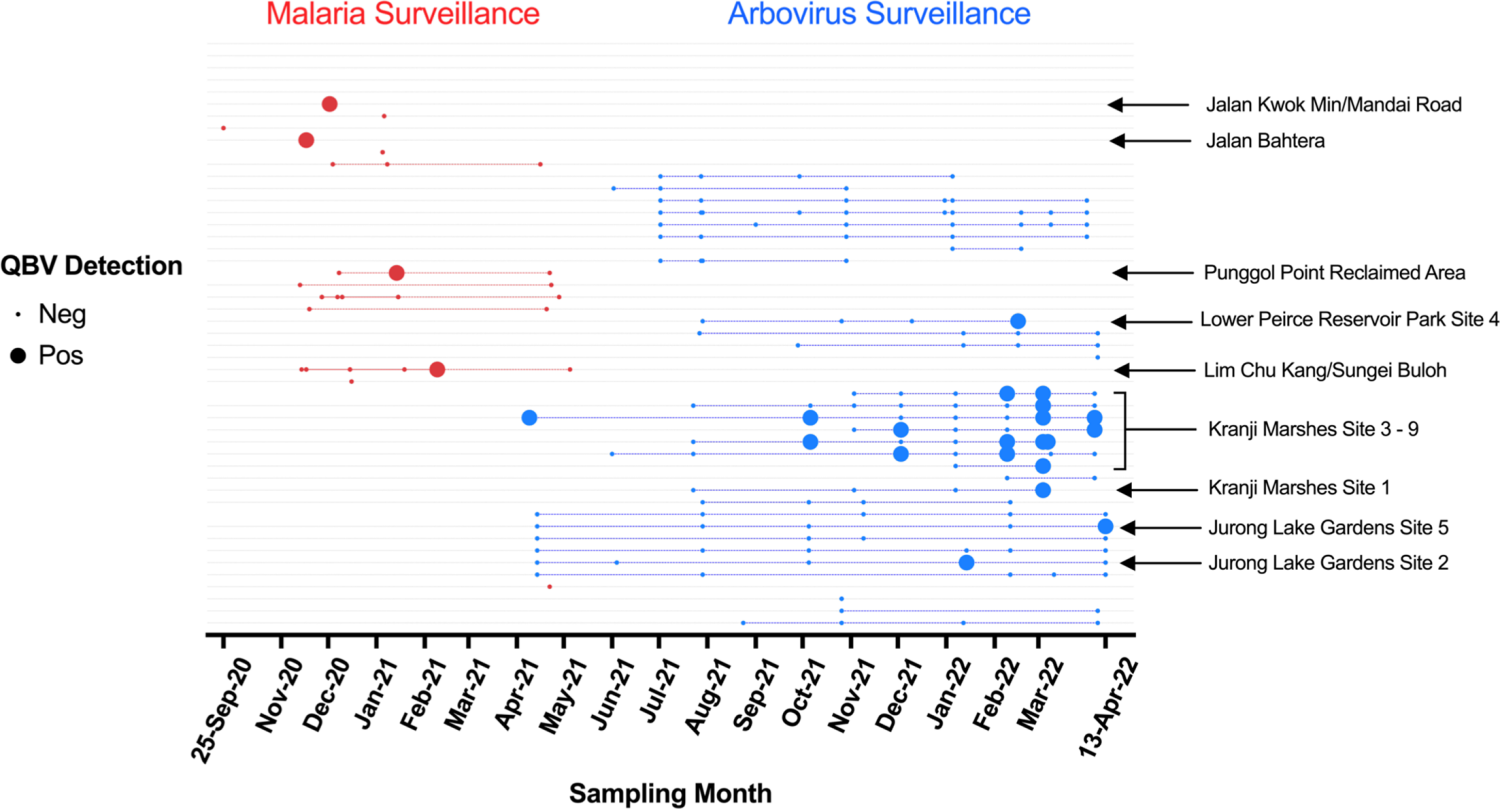
Spatiotemporal distribution of QBV in Singapore. Each small dot point represents an independent sampling event, while sites sampled repeatedly over time are connected by coloured dotted lines. Larger dots represent a positive QBV detection. Sampling events from malaria and arbovirus surveillance networks are denoted in red and blue, respectively. Sites sampled are arranged vertically, with sites containing QBV detections indicated on the right.

From the 721 pools, 36 pools were RT-qPCR positive for QBV across 7 sampling locations throughout Singapore (Table 2). Majority of the detections were located along north coastal sampling locations (32 pools), while the remaining pools were dispersed within central Singapore (Fig. 1a). From the 32 QBV-positive pools located along the northern coast of Singapore, 29 pools originated from nature reserves (Table 2), with repeated detection of QBV from 5 trapping sites within 1 of the nature reserves (Fig. 2). The remaining QBV RT-qPCR-positive pools were sporadic, single detections across Singapore from various nature reserves and forested areas (Fig. 1a and Table 2). QBV was also detected from various mosquito genera, namely, *Culex*, *Verrallina*, *Aedes* and *Aedeomyia*, with a majority of pools coming from *Culex* mosquitoes (27 pools) (Table 2). While QBV was only detected in a single species from *Verrallina*, *Aedes* and *Aedeomyia* mosquitoes, the virus was detected in *Cx. gelidus*, *Cx. nigropunctatus*, *Cx. quinquefasciatus*, *Cx. sitiens*, *Cx. tritaeniorhynchus* and *Cx. vishnui* (Table 2). It should be noted that QBV was detected in another seven pools of *Culex* mosquitoes of unidentified species.

**Table 2.** List of QBV RT-qPCR detections from September 2020 to April 2022

Mosquito species	Pool ID	Mosquito per pool	Date collected	Sampling location	Partial NS5 sequence	GenBank accession no.	Remark
*Cx. tritaeniorhynchus*	CT22_06	100	9 February 2022	Kranji Marshes	Y	PV010631	Nature reserve
*Cx. sitiens*	CS22_16	33	4 March 2022	Kranji Marshes	Y	PV010632	Nature reserve
*Cx. tritaeniorhynchus*	CT22_35	33	7 March 2022	Kranji Marshes	Y	PV010633	Nature reserve
*Cx. tritaeniorhynchus*	CT21_127	46	6 October 2021	Kranji Marshes	Y	PV010634	Nature reserve
*Cx. gelidus*	490 Cx. ge 9/2/21 T1	30	9 February 2021	Lim Chu Kang/Sungei Buloh	Y*	PV010628	Nature reserve
*Cx. gelidus*	490 Cx. ge 9/2/21 T5	30	9 February 2021	Lim Chu Kang/Sungei Buloh	Y*	PV010629	Nature reserve
*Cx. gelidus*	490 Cx. ge 9/2/21 T6	30	9 February 2021	Lim Chu Kang/Sungei Buloh	Y*	PV010630	Nature reserve
*Cx. tritaeniorhynchus*	283 Cx. Tri 2/12/20 T2	30	2 December 2020	Jalan Kwok Min/Mandai Rd	Y	PV010635	–
*Cx. sitiens*	CS22_09	1	14 January 2022	Jurong Lake Gardens	N	–	–
*Cx. tritaeniorhynchus*	CT22_32	100	4 March 2022	Kranji Marshes	N	–	Nature reserve
*Cx. vishnui*	CV22_22	17	9 February 2022	Kranji Marshes	N	–	Nature reserve
*Cx. vishnui*	CV22_29	15	4 March 2022	Kranji Marshes	N	–	Nature reserve
*Culex* spp.	CX22_13	90	4 March 2022	Kranji Marshes	N	–	Nature reserve
*Culex* (*Lophoceraomyia*) spp.	CX22_19	1	9 February 2022	Kranji Marshes	N	–	Nature reserve
*Culex* (*Lophoceraomyia*) spp.	CX22_23	89	4 March 2022	Kranji Marshes	N	–	Nature reserve
*Cx. sitiens*	CS22_15	5	9 February 2022	Kranji Marshes	N	–	Nature reserve
*Aedes albopictus*	AD22_6	2	16 February 2022	Lower Peirce Reservoir Park	N	–	Nature reserve
*Aedes albopictus*	AD22_8	3	4 March 2022	Kranji Marshes	N	–	Nature reserve
*Verrallina butleri*	AD22_18	100	4 March 2022	Kranji Marshes	N	–	Nature reserve
*Verrallina butleri*	AD22_20	80	4 March 2022	Kranji Marshes	N	–	Nature reserve
*Verrallina butleri*	AD22_25	100	4 March 2022	Kranji Marshes	N	–	Nature reserve
*Verrallina butleri*	AD22_28	70	4 March 2022	Kranji Marshes	N	–	Nature reserve
*Verrallina butleri*	AD22_29	77	4 March 2022	Kranji Marshes	N	–	Nature reserve
*Culex* (*Lophoceraomyia*) spp.	CX21_93	1	3 December 2021	Kranji Marshes	N	–	Nature reserve
*Culex* (*Lophoceraomyia*) spp.	CX21_94	5	3 December 2021	Kranji Marshes	N	–	Nature reserve
*Cx. vishnui*	CV22_41	19	6 April 2022	Kranji Marshes	N	–	Nature reserve
*Cx. sitiens*	CS22_30	5	13 April 2022	Jurong Lake Gardens	N	–	–
*Cx. quinquefasciatus*	CQ22_06	7	4 March 2022	Kranji Marshes	N	–	Nature reserve
*Cx. nigropunctatus*	CX21_84	1	9 April 2021	Kranji Marshes	N	–	Nature reserve
*Cx. nigropunctatus*	CX21_87	1	6 October 2021	Kranji Marshes	N	–	Nature reserve
*Culex* (*Lophoceraomyia*) spp.	CX22_38	1	6 April 2022	Kranji Marshes	N	–	Nature reserve
*Culex* (*Lophoceraomyia*) spp.	CX22_42	1	6 April 2022	Kranji Marshes	N	–	Nature reserve
*Aedeomyia catasticta*	216 A. cata 17/11/20 T1	30	17 November 2020	Jalan Bahtera	N	–	–
*Aedeomyia catasticta*	216 A. cata 17/11/20 T2	30	17 November 2020	Jalan Bahtera	N	–	–
*Cx. tritaeniorhynchus*	397 Cx. tri 14/1/21 T6	30	14 January 2021	Punggol Point Reclaimed Area	N	–	–
*Cx. tritaeniorhynchus*	397 Cx. tri 14/1/21 T40	30	14 January 2021	Punggol Point Reclaimed Area	N	–	–

*Samples with full genome sequences.

### Phylogenetic and molecular analysis of QBV

**Fig. 3.**
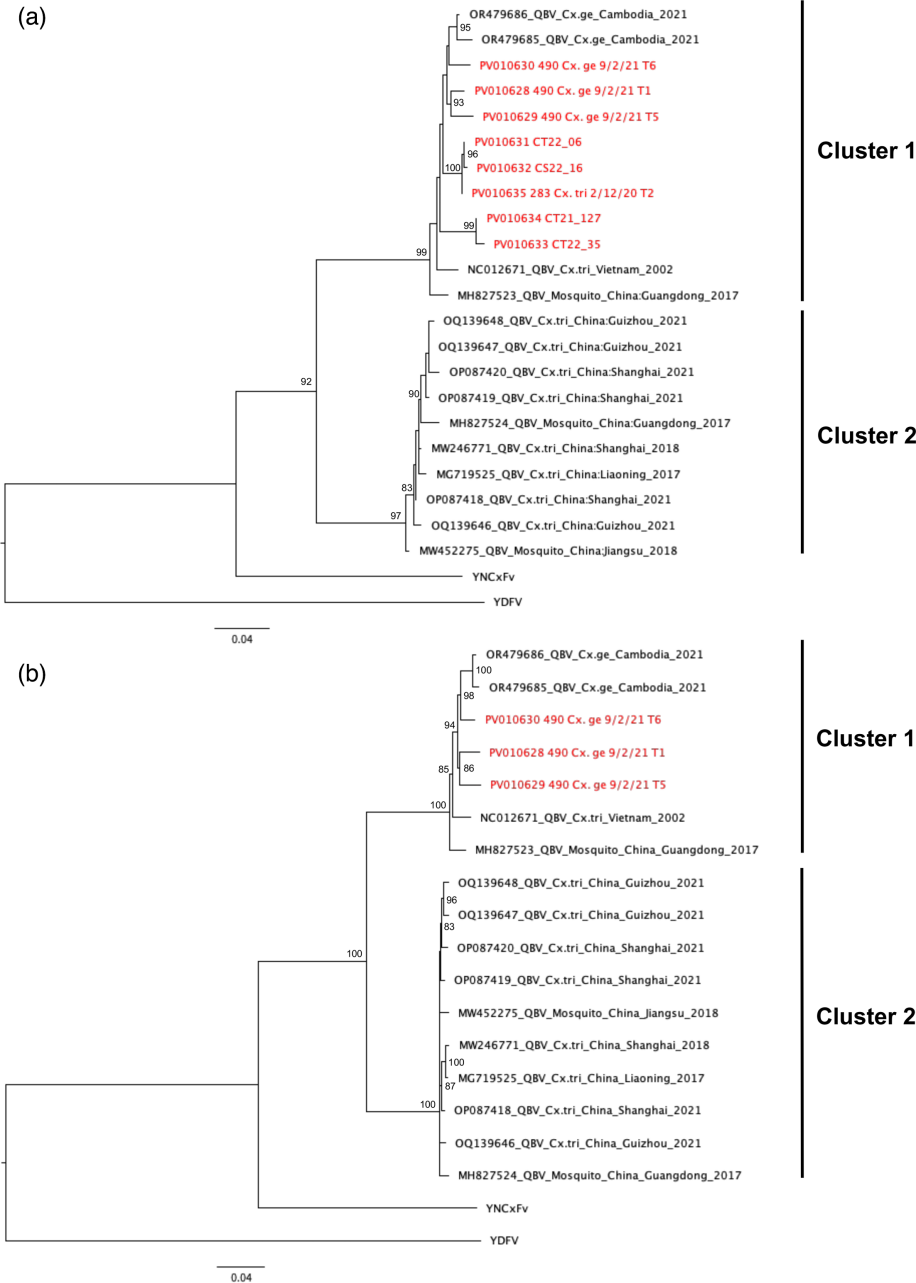
Phylogenetic trees of QBV from Singapore. Maximum likelihood phylogenetic trees constructed from (**a**) partial NS5 (533 bp) and (**b**) complete coding (10,080 bp) sequences of QBV. Bootstrap values below 80 are not shown. Sequences obtained from this study are highlighted in red.

**Fig. 4.**
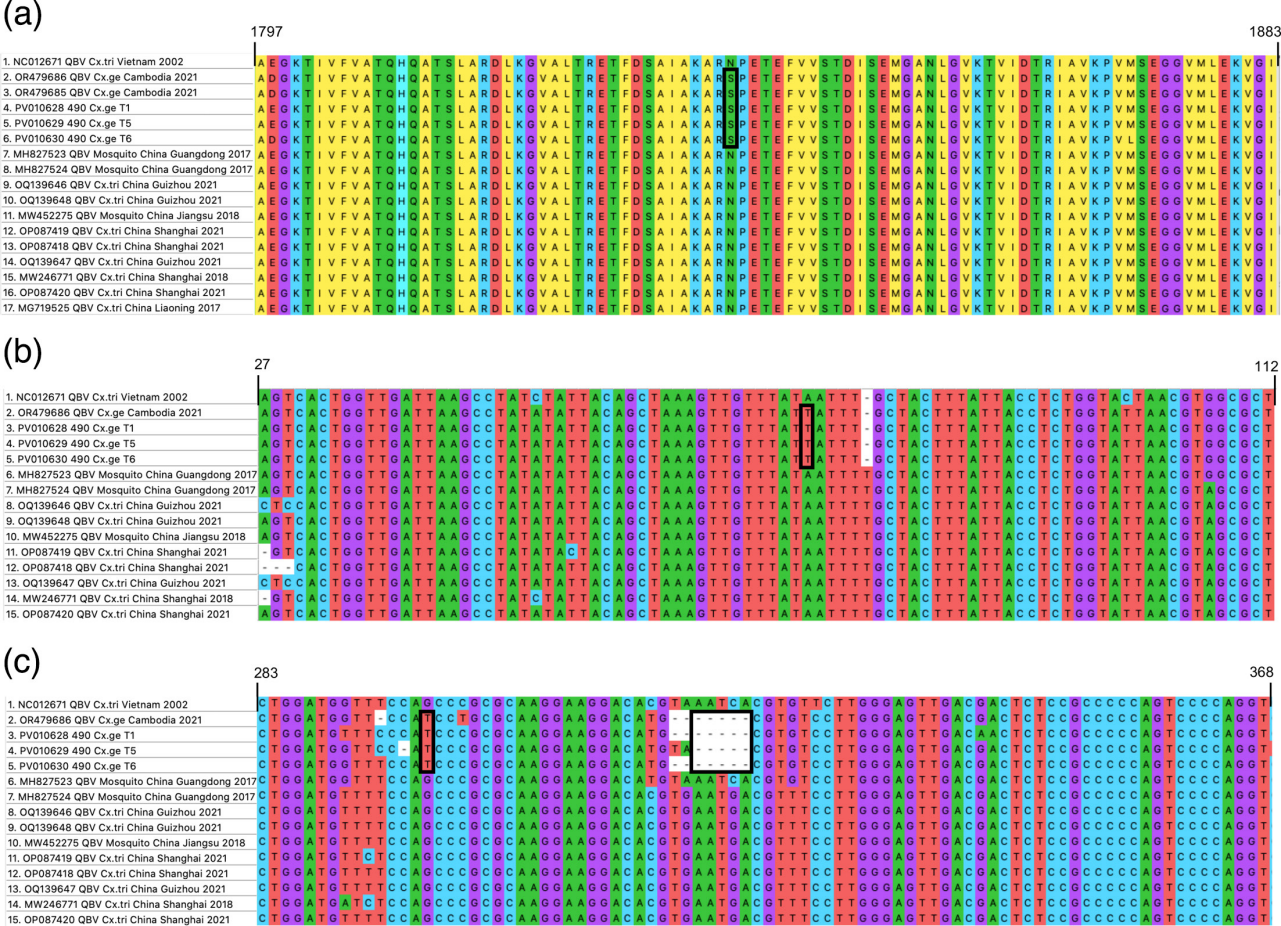
Species-specific mutations of QBV. (**a**) aa, (**b**) 5′ UTR and (**c**) 3′ UTR alignments of QBV sequences. Mutations found only in *Cx. gelidus*-derived sequences are in boxes.

From the 36 QBV RT-qPCR-positive mosquito pools, partial QBV NS5 sequences for phylogenetic analysis were successfully obtained from 8 pooled samples using the QBV_seq_1F/QBV_seq_1R primer designed for this study (Table 2). Following this, whole-genome sequencing using a panel of primers was conducted on these pools, resulting in three complete QBV genome sequences (Table 2). The partial NS5 and complete coding sequences of QBV obtained were used to construct two phylogenetic trees (Fig. 3). Previous studies have grouped QBV into two distinct clusters [54,55]. Cluster 1 consists of Southeast Asian sequences from Vietnam and southern China, while Cluster 2 consists of east Asian sequences from south-eastern and eastern China. All eight partial NS5 sequences obtained from this study group together with Vietnam and Guangdong sequences in Cluster 1 (Fig. 3a). Within Cluster 1, Southeast Asian (Vietnam, Singapore and Cambodia) partial NS5 and complete coding sequences are phylogenetically distinct from the Guangdong sequence, with *Cx. gelidus*-derived sequences forming a group within Cluster 1 (Fig. 3a, b). Furthermore, the full genome sequences of QBV revealed 67 non-synonymous mutations in all, except prM, viral genes that differentiate Cluster 1 and Cluster 2 QBV (Table S4). Only one of the non-synonymous mutations (NS3-N1837S) is specific to QBV sequences obtained from *Cx. gelidus* mosquitoes (Fig. 4a). In the 5′ and 3′ UTRs, two single nucleotide polymorphisms (SNPs) and a five-nt deletion were unique to *Cx. gelidus*-derived QBV sequences (Fig. 4b, c).

### *In vitro* characterization and superinfection exclusion potential of QBV

**Fig. 5.**
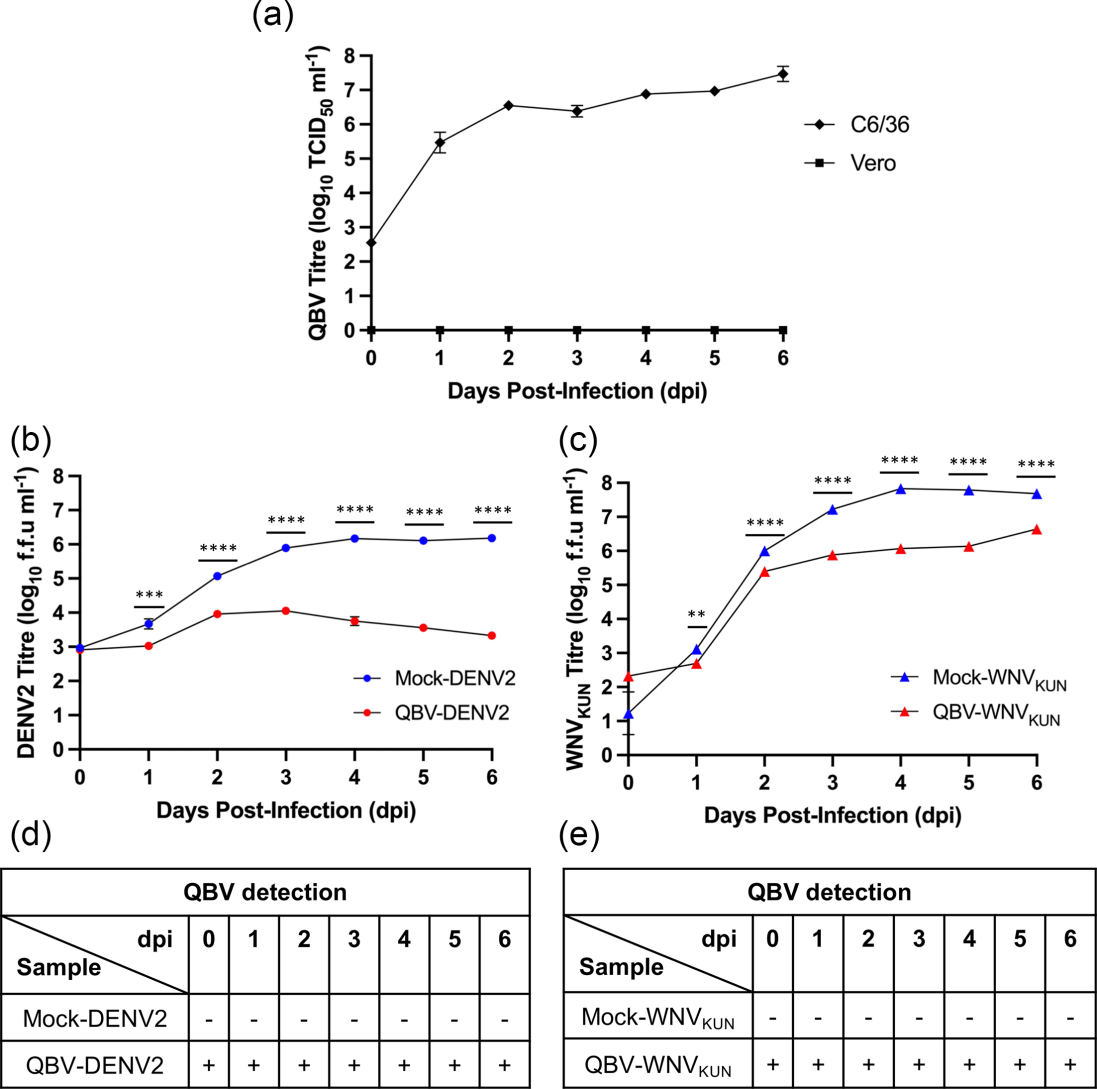
*In vitro* characterization and superinfection exclusion potential of QBV. (**a**) Growth kinetics of QBV in C6/36 and Vero cells infected at an m.o.i. of 0.1. Viral titres of (**b**) DENV2 and (**c**) WNV_KUN_ obtained from the supernatant of C6/36 cells after primary infection with QBV at an m.o.i. of 1 and subsequent infection with DENV2 or WNV_KUN_ at an m.o.i. of 0.1. Stable infection of cells with QBV prior to (**d**) DENV2 and (**e**) WNV_KUN_ was confirmed via RT-qPCR. Each datapoint represents the mean titre from biological triplicates with error bars showing the sem. Statistical significance was determined using two-way ANOVA followed by Tukey’s test. **P<0.01, ***P<0.001, ****P<0.0001.

QBV isolated from *Cx. gelidus* (Quang Binh virus isolate 490 Cx. ge 9/2/21 T1) was chosen for further *in vitro* experiments. The isolate grows efficiently in C6/36 cells, reaching 4.29×10^6^ TCID_50_/ml within 2 days post-infection (dpi) and 3.67×10^7^ TCID_50_/ml after 6 dpi, but did not grow in Vero cells (Fig. 5a). To determine if QBV could modulate the replication of medically important orthoflaviviruses, C6/36 cells were first infected with QBV, at an m.o.i. of 1, 1 h prior to secondary infection with either DENV2 or WNV_KUN_ (Fig. 5b, c, respectively). Stable infection of QBV was confirmed with RT-qPCR for all time points (Fig. 5d, e). QBV-infected cells exhibited significant inhibition of both DENV2 and WNV_KUN_ from 1 dpi, with viral titres reduced by up to 2.9 log and 1.8 log at 6 dpi and 4 dpi, respectively. In mock-infected controls, medically important orthoflaviviruses grew exponentially at 1 dpi before plateauing from 4 dpi onwards. When superinfected with QBV, both DENV2 and WNV_KUN_ viral titres plateaued abruptly at 2 dpi. While DENV2 titres gradually declined after 3 dpi, WNV_KUN_ titres started to recover after 5 dpi. In both experiments, cytopathic effects were observed starting at 3 dpi and progressed gradually until the end of the experiment.

## Discussion

Since the discovery of QBV in 2009, detections of the lineage I ISF have been reported in Southeast Asia and various regions of China [12,53,57]. To date, QBV has been reportedly isolated from *Cx. tritaeniorhynchus* mosquitoes in Vietnam and China [12,56]. However, none of those studies investigated the potential of QBV as a biological control against medically important viruses. Using a previously established cell-based screening assay, we report the isolation of QBV from three pools of *Cx. gelidus* mosquitoes in a north-western Singapore nature reserve [60,61]. This marks the first isolation of an ISF in Singapore and the first isolation of QBV from *Cx. gelidus* mosquitoes.

We also report the detection of QBV via RT-qPCR in seven rural locations in Singapore across an 18-month surveillance period. The majority (29/36 pools) of these detections were from nature reserves located in north-western and central Singapore, with 25 positive pools originating from *Culex* spp., *Aedes* sp. and *Verrallina* sp. mosquitoes in Kranji Marshes (Fig. 1a, Table 2). Five sampling sites within Kranji Marshes showed recurrent detection of QBV compared to sporadic, single detections from the other sampling locations. The highest number of QBV detections (13 positive pools) occurred in March 2022, coinciding with a peak in mosquito samples, mainly contributed by trappings from Kranji Marshes (Figs1b  2). This is in contrast to January 2021, which had the greatest number of mosquitoes collected, mainly contributed by Pasir Ris/Sungei Serangoon, yielding no QBV detections (Fig. S1). This suggests that there is a stable establishment of QBV in Kranji Marsh, albeit at a low prevalence (25/189 pools). Previous studies have also highlighted the low, yet persistent, prevalence of QBV in various regions of China [53,54]. As such, more research should be done to determine how QBV is maintained in nature despite the low infection rate in mosquitoes.

Thus far, QBV has mainly been detected in *Culex* spp. mosquitoes, namely, *Cx. tritaeniorhynchus, Cx. pipiens* and *Cx. gelidus*, with a few reports also detecting QBV in *Aedes vexans* and *An. sinensis* mosquitoes [54,55]. In this study, QBV RT-qPCR screening expanded the detection of QBV in additional *Culex* species including *Cx.* (*Lophoceraomyia*) spp., *Cx. nigropunctatus*, *Cx. quinquefasciatus*, *Cx. sitiens* and *Cx. vishnui* and further expanded the host range of QBV to *Verrallina butleri*, *Aedes albopictus* and *Aedeomyia catasticta*. However, it is important to note that the wide range of reported QBV hosts is based solely on RT-qPCR results. Similar molecular screenings for the lineage I ISFs, Aedes flavivirus and Hanko virus, report a wide host range of more than one mosquito species [85,86]. However, *in vitro* isolation of these viruses was only successful from a single mosquito species, suggesting an exaggeration of ISF host range when relying exclusively on molecular methods [14,16, 86,91]. As such, previous isolations from *Cx. tritaeniorhynchus*, and isolations from *Cx. gelidus* in this study, may be more representative of QBV’s host range [12,56]. Future *in vitro* and *in vivo* experiments in a range of mosquito genera would provide stronger evidence for the host range of QBV.

Our phylogenetic analysis reveals that all partial NS5 and complete coding sequences of QBV obtained from Singapore group with other Southeast Asian sequences in Cluster 1, and are phylogenetically distinct from the Chinese sequences in Cluster 2. This further supports the notion of geographical segregation between Cluster 1 and Cluster 2 QBV suggested by Fang *et al.*, with Guangdong being the boundary separating the two QBV clusters [54,55]. Since all QBV detections have been localized in China and Southeast Asia, it would be interesting to determine the directionality of virus evolution within the region using phylogeography. However, the small number of available full genome sequences would make such analysis unreliable. Interestingly, *Cx. gelidus*-derived sequences were phylogenetically distinct from *Cx. tritaeniorhynchus*-derived sequences in Cluster 1. Upon analysis of full genome sequences, we identify an NS3-N1837S mutation, two UTR SNPs and a five-nt 3′ UTR deletion that are specific to sequences obtained from *Cx. gelidus* mosquitoes. More work is needed to ascertain the effects of these mutations in different mosquito species.

In this study, we report QBV’s ability to modulate secondary infections by DENV2 and WNV_KUN_ *in vitro* via superinfection exclusion. While this provides promising signs to the potential use of QBV as a biological control, *in vivo* data is still crucial for several reasons. Results obtained from C6/36 cells used in this study might not be representative of *in vivo* infection as C6/36 cells are RNA interference deficient, a critical antiviral response system in mosquitoes [92]. For instance, *in vitro* superinfection exclusion of WNV by Nhumirim virus (NHUV) and *Culex* flavivirus (CxFV) was not recapitulated in *Cx. pipiens* and *Cx. quinquefasciatus* mosquitoes, respectively [43,44]. Previous studies have demonstrated that establishing laboratory colonies infected with an ISF presents a complex challenge. Hall-Mendelin *et al*. highlighted that *in vivo* Palm Creek virus (PCV) infection was only successful via intrathoracic injection and not infectious bloodmeal for *Culex annulirostris*, suggesting midgut barriers to ISF infection [39]. Additionally, both Hall-Mendelin *et al*. and Koh *et al*. showed that PCV could not be vertically transmitted in *Cx. annulirostris* and *Aedes aegypti* mosquitoes [38,39]. In contrast, transmission routes of CFAV and CxFV have been shown to be vertical and venereal [43,93]. Previous studies have also shown species-specific host range restriction of ISFs, further complicating *in vivo* infection [28]. The complexities involving ISF infection, superinfection exclusion and transmission in mosquitoes highlight the importance of *in vivo* studies with QBV.

Currently, Singapore has been successful in using a *Wolbachia*-mediated population suppression strategy, involving the release of male *Wolbachia*-infected mosquitoes, to suppress the local *Aedes aegypti* population and reduce the risk of dengue incidence [94,95]. This poses a question to the usefulness of ISFs as a biological control agent and their potential interactions with *Wolbachia*-infected mosquitoes. Previous *in vitro* and *in vivo* studies have shown that *Wolbachia* generally downregulates +ssRNA virus infection [96]. Interestingly, Amuzu *et al*. presented confounding evidence of increased ISF infection in *wMel*-infected *Aedes aegypti* [97]. Nonetheless, the potential reduction of ISF infection in the presence of *Wolbachia* suggests that both should not be used in tandem when associated with the same genera of mosquitoes. However, given QBV’s natural association with *Culex* mosquitoes and significant reduction of WNV_KUN_ infection *in vitro*, an ISF-mediated biological control in *Culex* mosquitoes could be used in parallel with *Wolbachia*-infected *Aedes aegypti* releases to combat other medically important orthoflaviviruses. While previous studies have shown that CFAV does not incur any fitness cost in *Aedes aegypti* mosquitoes, studies with QBV will need to be conducted to determine its effect on mosquito fitness [98].

In conclusion, we report the first isolation of the ISF, QBV, from *Cx. gelidus* mosquitoes in Singapore and its spatiotemporal prevalence in Singapore. We also present *in vitro* evidence that QBV is able to suppress secondary infections of medically important orthoflaviviruses. Future *in vivo* experiments are required to determine the transmission and biological applications of the virus.

## Supplementary material

10.1099/jgv.0.002105 Fig. S1.

10.1099/jgv.0.002105 Supplementary Material 1.

## Abbreviations

BHK: baby hamster kidney
CFAV: cell-fusing agent virus
CxFV: *Culex* flavivirus
DENV: dengue virus
dpi: days post-infection
ISF: insect-specific flavivirus
ISV: insect-specific virus
JEV: Japanese encephalitis virus
MAVRIC: monoclonal antibodies against viral RNA intermediates in cells
NEA: National Environment Agency
NGS: next-generation sequencing
PBST: phosphate-buffered saline containing 0.05 % v/v Tween 20
PCV: Palm Creek virus
QBV: Quang Binh virus
RT: reverse transcription
RT-PCR: reverse transcriptase PCR
RT-qPCR: reverse transcriptase quantitative PCR
SISPA: sequence-independent, single-primer amplification
SNP: single nucleotide polymorphism
+ssRNA: positive-strand ssRNA
VIF: vertebrate-infecting flavivirus
WNV: West Nile virus
YNCxFV: YunNan *Culex* flavivirus
