# Supplementary figures and images for: Spatiotemporal prevalence and characterization of the lineage I insect-specific flavivirus, Quang Binh virus, isolated from Culex gelidus mosquitoes in Singapore
Source: J Gen Virol. 2025 Jun 2;106(6):002105. doi: 10.1099/jgv.0.002105 (PMC12163729; doi:10.1099/jgv.0.002105)

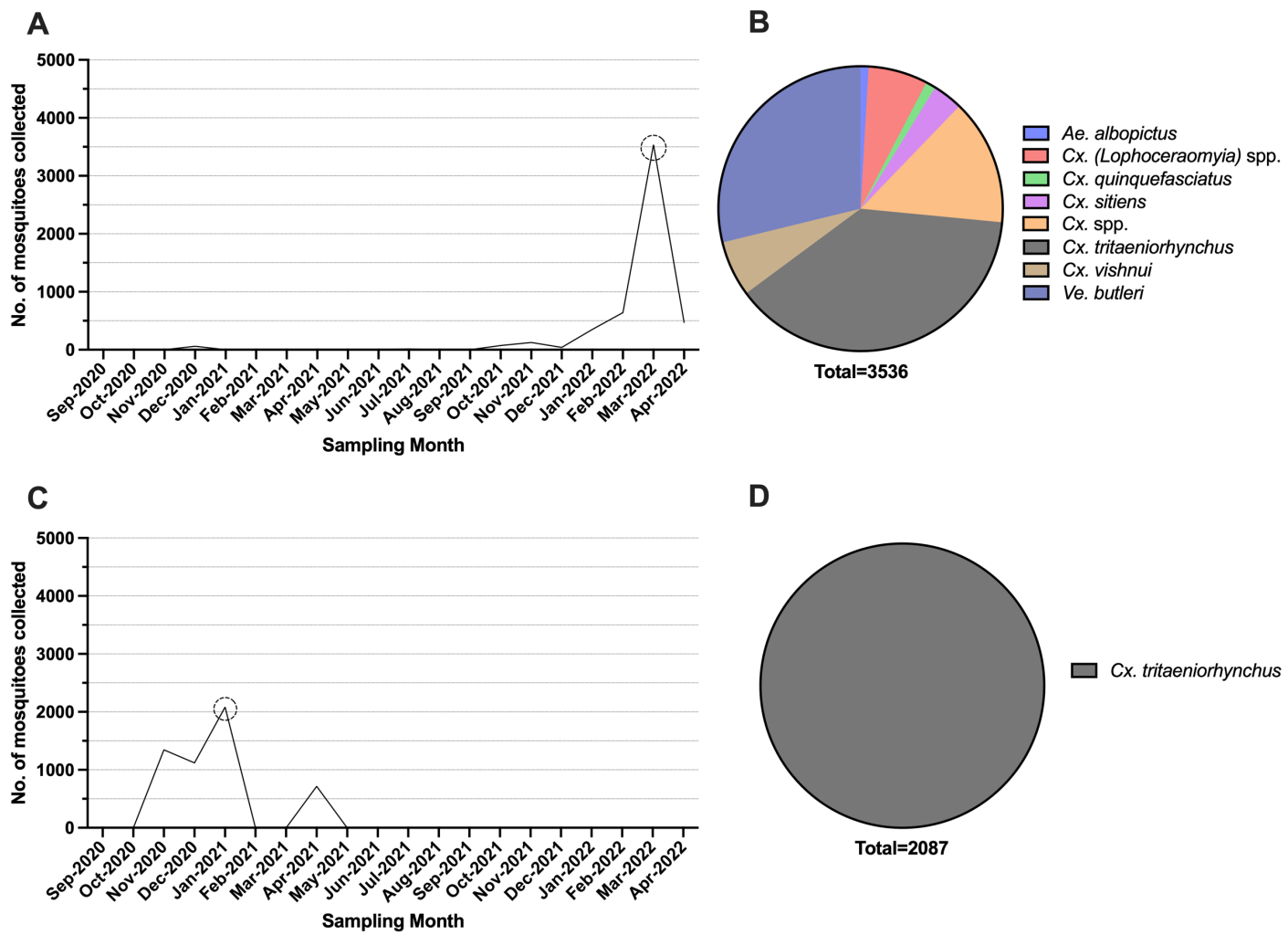

Supplement: Fig. S1. [file jgv-106-02105-s001.pdf]
